# The Sulphur Poisoning Behaviour of Gadolinia Doped Ceria Model Systems in Reducing Atmospheres

**DOI:** 10.3390/ma9080649

**Published:** 2016-08-02

**Authors:** Matthias Gerstl, Andreas Nenning, Riza Iskandar, Veronika Rojek-Wöckner, Martin Bram, Herbert Hutter, Alexander Karl Opitz

**Affiliations:** 1 Institute of Chemical Technologies and Analytics, TU Wien, Getreidemarkt 9/164-EC, Vienna A-1060, Austria; andreas.nenning@tuwien.ac.at (A.N.); herbert.hutter@tuwien.ac.at (H.H.); 2 Christian Doppler Laboratory for Interfaces in Metal-Supported Electrochemical Energy Converters, Getreidemarkt 9/164-EC, Vienna 1060, Austria; Veronika.Rojek@plansee.com (V.R.-W.); m.bram@fz-juelich.de (M.B.); 3 Central Facility for Electron Microscopy (GFE), RWTH Aachen University, Ahornstraße 55, Aachen 52074, Germany; iskandar@gfe.rwth-aachen.de; 4 Plansee SE, Innovation Services, Reutte 6600, Austria; 5 Forschungszentrum Jülich GmbH, Institute of Energy and Climate Research, Materials Synthesis and Processing (IEK-1), Jülich 52425, Germany

**Keywords:** ceria, sulphur poisoning, SOFC anode, electrochemical impedance spectroscopy, ToF-SIMS, microelectrodes, XRD, XPS, BET, TEM

## Abstract

An array of analytical methods including surface area determination by gas adsorption using the Brunauer, Emmett, Teller (BET) method, combustion analysis, XRD, ToF-SIMS, TEM and impedance spectroscopy has been used to investigate the interaction of gadolinia doped ceria (GDC) with hydrogen sulphide containing reducing atmospheres. It is shown that sulphur is incorporated into the GDC bulk and might lead to phase changes. Additionally, high concentrations of silicon are found on the surface of model composite microelectrodes. Based on these data, a model is proposed to explain the multi-facetted electrochemical degradation behaviour encountered during long term electrochemical measurements. While electrochemical bulk properties of GDC stay largely unaffected, the surface polarisation resistance is dramatically changed, due to silicon segregation and reaction with adsorbed sulphur.

## 1. Introduction

Improving solid oxide fuel cells (SOFCs) to a competitive technology requires a joint effort in process engineering, manufacturing and materials science [1,2]. In the latter, important advances have been made with the introduction of high performing mixed ionic electronic conductors (MIEC) as cathode materials [1]. These perovskite-type materials like lanthanum strontium cobalt or cobalt iron oxide (LSC or LSCF) have succeeded the triple phase boundary active lanthanum strontium manganite as the electrode material of choice [3]. An analogous approach on the anode side of SOFCs may lead to similar improvements in performance. Gadolinia doped cerium oxide (GDC) exhibits MIEC properties in reducing atmospheres and, in combination with metallic current collectors, is considered a prime candidate as a future alternative anode material. Furthermore, it has been shown that infiltration of classical nickel/yttria stabilized zirconia (Ni/YSZ) ceramic-metal composite SOFC anodes with doped or undoped cerium oxide, or even complete substitution of YSZ, improves their resilience against sulphur poisoning [4,5,6,7,8,9,10,11,12,13,14]. This property is highly sought after for enabling the commercialization of SOFCs, where tolerance against hydrogen sulphide containing fuels like diesel reformate or bio gas is an important requirement. Recent reviews concerning this topic can be found in Refs. [15,16]. In the case of Ni/YSZ, it is generally accepted that the nickel phase is quickly completely covered with adsorbed sulphur, which also blocks the active triple phase boundary [15,17]. While this explanation seems reasonable, a recent study showed that despite the high sulphur coverage of the Ni phase, it retains its catalytic activity in large parts [11]. However, by introduction of the mixed conducting ceria, the reactive site is extended onto the ceria surface, making Ni/ceria based cermets less prone to sulphur poisoning [10,13].

Apart from sulphur poisoning on Ni, there are also studies, which also focus on the effects of H_2_S on ceria. For example, a SOFC using a novel Cu/ceria anode was reported to be almost insensitive to H_2_S concentrations of up to 450 ppm in the fuel. However, a slight drop in power density compared to sulphide free fuel gas can be gleaned from the text. Visible long term degradation sets in at concentrations of about 300 ppm H_2_S, which was associated with the formation of a cerium oxysulphide phase: Ce_2_O_2_S [15,18]. Studying copper containing ceria H_2_S sorbents, it was found that copper actually increases the bulk sulphur uptake of ceria and facilitates the reaction to cerium oxysulphide by reduction of Ce^4+^ to Ce^3+^ [19]. Indeed, this mechanism has been proposed to explain the reversible H_2_S poisoning of ceria infiltrated Ni/YSZ SOFC anodes, by ceria acting as a sulphur scavenger. Furthermore, it was found that anodically polarised electrodes show less degradation and recover faster than unpolarised ones [12]. Moreover, anodically polarised doped ceria electrodes catalytic properties toward H_2_S oxidation [12,20]. This might reconciliate the seemingly contradictory results published on the Cu/ceria system.

It seems like a logical next step to test Ni/GDC as a substitute for the traditional Ni/YSZ cermet electrodes, which show severe degradation under operation with sulphur containing fuels [10,13,16,21,22]. However, a thorough understanding of the poisoning mechanisms of both Ni/YSZ and GDC is important for an informed improvement strategy toward sulphur tolerant SOFC anodes [10,11,12,15,17,18,23,24].

In the present study, an array of analytical methods, including transmission electron microscopy (TEM), BET surface area analysis, X-ray diffraction (XRD), time of flight secondary ion mass spectrometry (ToF-SIMS), X-ray photoelectron spectroscopy (XPS), electrochemical impedance spectroscopy (EIS) and combustion analysis, is used to characterize different aspects of the interaction of hydrogen sulphide with GDC model systems in reducing atmospheres. This wealth of information is used to formulate a tentative model explaining the multi-facetted electrochemical degradation behaviour of GDC model composite electrodes, observed in long term electrochemical impedance spectroscopy studies.

## 2. Experimental

### 2.1. Preparation of the Model Composite Electrodes

**Figure 1:**
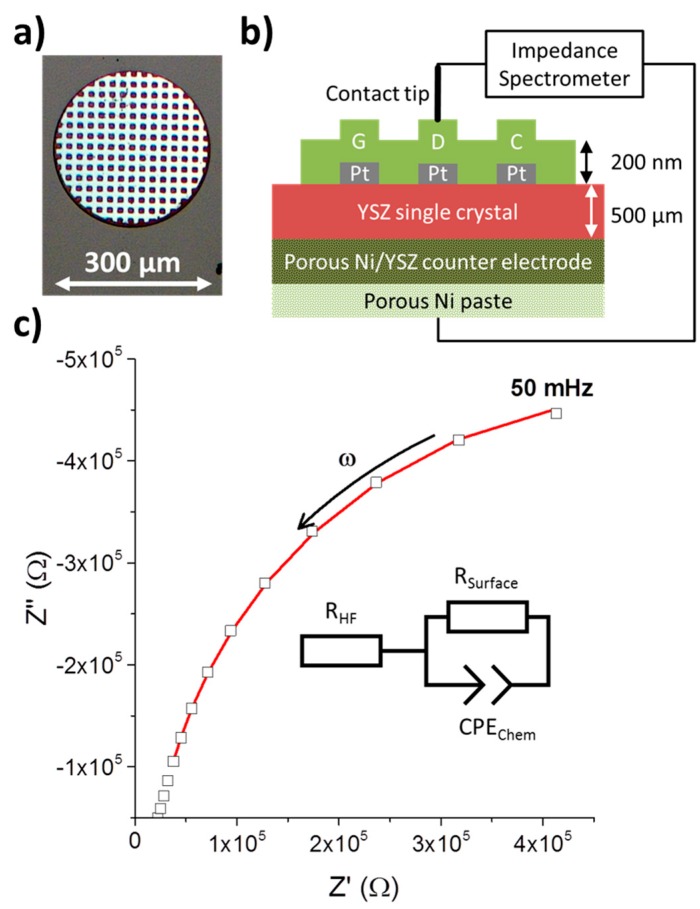
(**a**) Optical micrograph of a circular Pt/GDC 10 composite microelectrode. The bright grid is a 100 nm thick Pt current collector of 10 µm width and with 10 × 10 µm^2^ holes beneath a 200 nm GDC 10 thin film; (**b**) Schematic cross sectional view of a Pt/GDC microelectrode setup wired for impedance measurement; (**c**) Electrical impedance spectrum (squares) with data fitting (**red** line) to the equivalent circuit shown. The medium frequency part is not considered in the data fitting, see Section 2.11.

**Figure 2:**
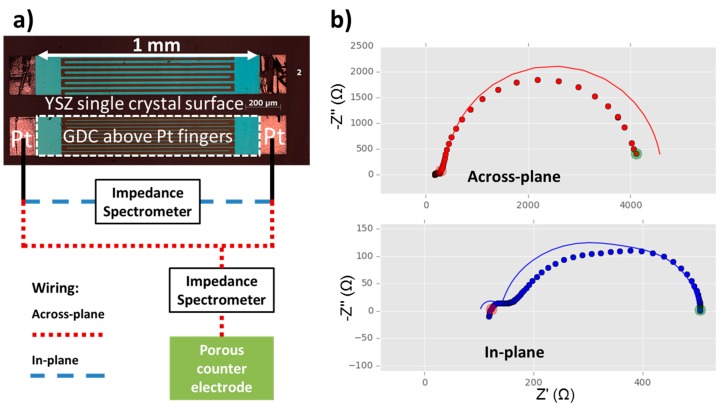
(**a**) Optical micrograph of GDC model composite microelectrodes (bright **blue** rectangles with dashed white frame) on top of buried interdigitated Pt current collectors with a circuit diagram for the measurements in across- and in-plane mode according to reference [25]. The Pt current collectors are slightly larger than the GDC electrode for easier contacting (bright **red** rectangles). The layer sequence and the placement of the counter electrode in the cross sectional view is analogous to Figure 1a; (**b**) Measured data—shown as dots—and fits to the equivalent circuit model according to reference [25]—shown as lines—for in- and across-plane measurements of a model composite microelectrode with buried Pt current collectors. Both spectra are fitted simultaneously by one and the same parameter set. The interdigitated current collectors had a width and spacing of both 30 µm and a length of 980 µm with a total of 12 digits. Measurements were performed at 750 °C in humidified ~2.5% vol H_2_ in Ar, with a ratio of hydrogen to water of 1:1.

Counter electrodes were prepared by manually screen printing Ni/YSZ paste (Fuel Cell Materials, Lewis Center, OH, USA) and subsequently Ni paste on the back side of polished YSZ (100) single crystalline 1 × 1 cm^2^ substrates with a thickness of 0.5 mm purchased from Crystec, Berlin, Germany. The counter electrodes were fired at 300 °C for 30 min and sintered for 2 h at 1250 °C. Current collectors consisting of 100 nm thin Pt films on a 5 nm thick Ti adhesion layer were both deposited by magnetron sputtering (Bal-Tec, Balzers, Liechtenstein) onto the polished side of YSZ single crystals with already prepared counter electrodes. Micro-patterning of the current collectors was done using photolithography with subsequent Ar ion beam etching in a vacuum chamber. Afterwards, GDC layers of 200 nm thickness were prepared by pulsed laser deposition (PLD) on top of these samples with current collectors. PLD-targets were prepared from 10% or 20% Gd_2_O_3_ doped CeO_2_ (Treibacher, Althofen, Austria), for the sake of brevity called GDC 10 and GDC 20, respectively. The rationale behind the choice of two different doping levels was to use the less degradation prone GDC 10 for electrochemical measurements, while the higher concentration of oxygen vacancies in GDC 20 was deemed beneficial for ion exchange experiments. However, in practice the difference between the two materials turned out to be negligible. The deposition took place at 0.04 mbar oxygen background pressure and the substrate temperature was usually about 750 °C, controlled by a pyrometer (Heitronics, Wiesbaden, Germany). A Lambda COMPexPro 201F KrF excimer laser (Coherent, Santa Clara, CA, USA) operating at λ = 248 nm with a pulse frequency of 5 Hz and a pulse length of 50 ns was used to ablate the GDC target with an approximate fluence of about 1.5 J·cm^−2^ on the target. The distance between target and substrate was fixed at 6 cm. Micro-patterning of the GDC layer was also done by photolithography and ion beam etching. Two types of model composite electrodes were prepared in this manner: circular GDC electrodes with a grid shaped Ti/Pt current collector, see Figure 1, and rectangular GDC electrodes with two interdigitated Ti/Pt current collectors, see Figure 2.

### 2.2. Preparation of the Porous Ni/GDC Electrodes

The porous Ni/GDC cermet electrodes were prepared by screen printing on both sides of a polycrystalline YSZ electrolyte. Details of the process are given in reference [26].

### 2.3. Preparation of Macroscopic Polycrystals

Fine GDC 10 powder (Treibacher, Althofen, Austria) was cold isostatically pressed and sintered at 1350 °C for 5 h with heating and cooling ramps of 2 °C per minute in ambient air. The sintered polycrystals were cut to about 1.5 mm thickness and automatically polished down to a 1 µm diamond paste finish (Struers, Ballerup, Denmark).

### 2.4. BET and Combustion Analysis

The specific surface area of powder samples was determined using automated BET adsorption (Areamat, Jung, Viersen, Germany). The sulphur content of the powder samples was determined by combustion analysis and consecutive infrared detection and quantification in a Leco CS 600 analyser (San Jose, CA, USA).

### 2.5. X-ray Diffraction

Gracing incidence X-ray diffraction measurements were performed on a PANalytical Empyrean machine (PANalytical, Almelo, The Netherlands) equipped with a Cu tube. On the primary beam path, a parallel beam mirror was used with a 0.04 rad soller collimator and a 0.5° divergence slit. On the secondary side, a parallel plate collimator was used and a PiXcel detector in 0d mode. The scans were taken with a fixed incidence angle of 2° from 10° to 100° with a step size of 0.03° in a continuous mode with counting time 0.484 s/point. The measurements were repeated six times and summed later with the HighScore software (PANalytical, Almelo, The Netherlands) [27].

### 2.6. Scanning Transmission Electron Microscopy

The specimens were analysed using scanning transmission electron microscopy (STEM). The cross-sectional STEM samples were prepared using a focused ion beam (FIB) technique on a Strata 205 FIB workstation (FEI, Hillsboro, OR, USA). The STEM investigations using energy-dispersive X-ray spectroscopy (EDS) analysis were performed on a field emission gun Zeiss Libra 200FE (Carl Zeiss, Oberkochen, Germany) operated at 200 kV equipped with in-column corrected omega filter, an X-Flash Energy Dispersive X-ray (EDX) detector (Bruker, Billerica, MA, USA) and a high-angle annular dark-field, HAADF, detector (Fischione, Export, PA, USA). The EDS semi-quantitative analysis was done using ESPRIT software from Bruker (Billerica, MA, USA).

### 2.7. Gases

Premixed gases of ca. 2.5% H_2_ in Ar (ARCAL 10) and 200 ppm H_2_S in ARCAL 10 were bought from Air Liquide (Paris, France) in purities better than 99.99%. Dilution of the concentrated hydrogen sulphide carrying gas was realized by analogue mass flow controllers. Except stated otherwise, the H_2_S free ARCAL 10 was bubbled through deionized water at room temperature to achieve a water to hydrogen ratio of about 1:1.

### 2.8. Diffusion Experiments

Concentration profiles of sulphur species in GDC 10 were measured using a TOF-SIMS V machine (IONTOF, Muenster, Germany) in high current bunched mode, see e.g., [28,29]. The ^34^S signal was used for evaluations on GDC thin film samples and normalized to the total secondary ion count to enable comparison between different measurements.

The concentration profiles of samples exposed to a tracer species for a specified time can be described by Equation (1) [30,31]:

(1) $$\frac{c\left(x,t\right)}{c_{gas}-c_{bg}}=erfc\left(\frac{x}{2\sqrt{D\times t}}\right)-e^{\left(h\cdot x+h^{2}D\cdot t\right)}\times erfc\left(\frac{x}{2\sqrt{D\times t}}+h\sqrt{D\times t}\right)$$

Symbol *c* in Equation (1) denotes the relative concentration of sulphur in the gas phase (tracer fraction), *c_bg_* is the relative background concentration of the tracer, *c_gas_* is the relative tracer concentration of the gas environment, *x* is the distance from the surface and *t* is the diffusion time; *h = k*/*D* reflects the ratio of the surface exchange coefficient *k* and the diffusion coefficient *D*.

For macroscopic samples, the ^34^S signal was very low and the ^32^S data were evaluated instead. To overcome the overlap of the ^32^S with the much more intense O_2_ signal, two Pearson VII peak functions [32] were fitted to the profiles with a fixed spacing of 0.0177 u, i.e., the mass difference between the two ions [29,33]. The results agree admirably with the ^34^S signal, but with a dramatically decreased data scatter. The Pearson VII function was chosen empirically as it offered the best fit to the data.

### 2.9. X-ray Photoelectron Spectroscopy

XPS spectra were collected using a monochromated 100W SPECS micro-focus 350 X-ray source and an angle resolved PHOIBOS WAL analyser (SPECS, Berlin, Germany) with a detection angle of 20° to 80° relative to the surface normal. Prior to mounting, the samples were heated in air at 400 °C, and subsequently in vacuum at roughly 350 °C, to reduce the coverage with adventitious carbon. Binding energies were calibrated by fixing the still detectable adventitious carbon peak at 285 eV.

### 2.10. Electrochemical Impedance Spectroscopy

Impedance spectra were recorded using an Alpha-A high-performance frequency analyser (Novocontrol, Montabaur, Germany) in the frequency range of 1 MHz to 50 mHz with an AC signal of 10 mV root-mean-square. The model-composite electrode measurements were done in a symmetrically heated three-terminal micro-contact setup, as described in detail in reference [34].

### 2.11. Impedance Data Evaluation on Model-Composite Microelectrodes

While in reducing atmospheres, GDC exhibits, to a certain degree, electronic conductivity [35]; this property is not sufficient to homogenously polarise 200 nm thin film electrodes made from pure GDC of a diameter of more than a few microns. Therefore, to guarantee polarisation of the whole microelectrode surface, an additional Pt current collector is applied beneath the GDC layer analogous to reference [36], see Figure 1.

A typical measurement of an impedance spectrum of a 300 µm diameter electrode along with the equivalent circuit to fit the data is shown in Figure 1c. Only the low frequency part from about 1 Hz to 50 mHz was used to fit the data, which almost exclusively consists of the parallel connection of the surface polarisation resistance *R_surf_* with the chemical capacitance *C_chem_* [25,37,38]. The resulting surface resistance *R_surf_* was normalized to the surface area of the circular microelectrodes.

To account for slightly non-ideal behaviour, the chemical capacitance was modelled by a constant phase element *CPE*, with impedance

(2) $$Z_{CPE}=\frac{1}{CPE{\left(i\omega \right)}^{n}}$$

From the constant phase element, the chemical capacitance *C_chem_*, according to reference [39], can be extracted by

(3) $$C_{chem}={\left(R_{surf}^{1-n}CPE_{chem}\right)}^{\frac{1}{n}}$$

The medium frequency parts contain additional information, but those are not easily extracted with this type of electrode [25] and the medium frequency range is thus not used in the fitting procedure. To account for the high and medium frequency offset, a series resistance *R_HF_* is introduced.

A more sophisticated current collector geometry and equivalent circuit model also enables interpretation of the features that deviate from this very simple equivalent circuit model [25]. This measurement technique is based on impedance spectroscopy on MIEC electrodes with rather low electronic conductivity, by means of two comb-shaped, interdigitated current collectors. These interdigitated electrodes allow two different ways of wiring, leading to in-plane and across-plane current flow, respectively. A sketch of the measurement modes with an optical micrograph is shown in Figure 2a. The two resulting impedance spectra (see Figure 2b) allow the additional extraction of ionic and electronic conductivity of the GDC model-composite microelectrode. For an in depth treatment of the theory behind this novel technique, please consult the original publication in reference [25].

To evaluate the data a fitting routine was written in the Python programming language using the scipy [40] and lmfit packages [41]. It is essential that the two impedance spectra measured on one microelectrode are analysed simultaneously by a single parameter set. In Figure 2b, a typical simultaneous fit of an across- and an in-plane measurement is shown. The fitting is not perfect, most likely because of invalid simplifications in the fitting model caused by the larger electrode size than in the original reference [25]. However, the general trends are well captured and the results compare reasonably to data recorded on smaller, and hence more ideal, circular microelectrodes or literature values, see Table 1.

## 3. Results and Discussion

In the following Section 3, experimental results gathered on different GDC model systems by various analytical methods will be presented. These results will be used to explain the sulphur poisoning behaviour of model composite GDC microelectrodes encountered in electrochemical impedance measurements and will be summarized in Section 4.

### 3.1. Sulphur Diffusion Experiments

#### 3.1.1. Composite Microelectrodes

**Figure 3:**
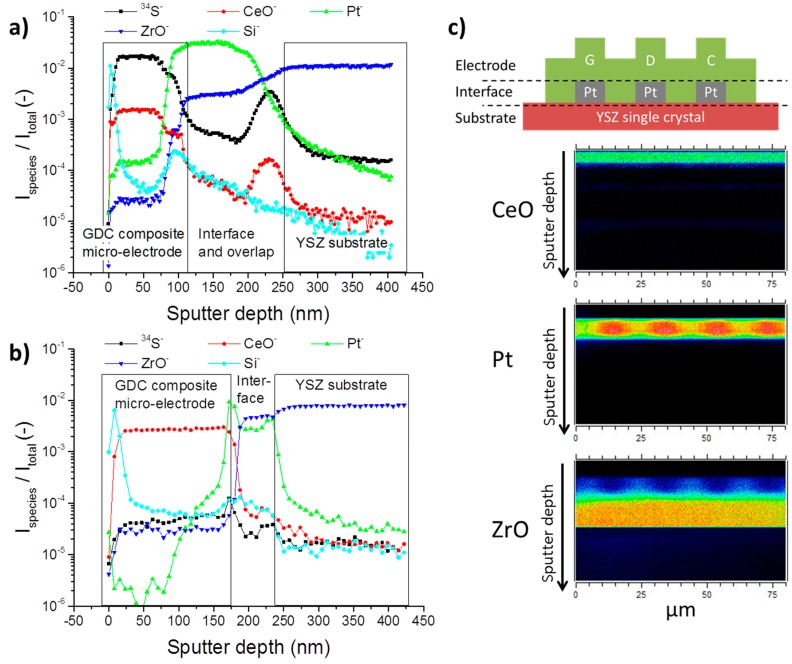
(**a**,**b**) ToF-SIMS depth profiles of Pt/GDC 10 composite microelectrodes after annealing in an atmosphere of 2.5% vol H_2_ in Ar with a hydrogen to water ratio of 1:1 and 10 ppm H_2_S for 22 h at 750 °C. (**a**) The electrode was additionally cathodically polarised at 1 V; (**b**) Depth profile of an unpolarised neighbouring electrode. The recorded signals of the different species were normalized to the total signal count to allow comparison between measurements; (**c**) Schematic visualization of the assignment to electrode, interface and substrate region in the depth profiles in (**a**,**b**) accompanied by cross sectional ion images of CeO, Pt and ZrO.

In Figure 3a, a ToF-SIMS depth profile of a GDC composite microelectrode is shown, which has been cathodically polarised at −1 V in an atmosphere of humidified hydrogen (H_2_:H_2_O ≈ 1:1) with 10 ppm H_2_S for 22 h at 750 °C. These rather harsh conditions were chosen to check whether or not sulphur can be accommodated at an appreciable amount in the GDC lattice from the gas phase and if a diffusion profile can be detected [42]. As a comparison, Figure 3b shows a depth profile of an unpolarised neighbouring electrode. For better orientation, the depth profiles in Figure 3 are divided in three parts roughly corresponding to the GDC layer of the composite microelectrode, the interface region where the Pt current collector is located and the YSZ substrate. These regions are also visualized in Figure 3c with the aid of a schematic view of the electrode and representative ion images.

The polarised electrode in Figure 3a shows that the ^34^S signal is increased by more than three orders of magnitude when compared to the reference measurement in Figure 3b. This result impressively shows that sulphur indeed can be incorporated into GDC electrochemically. The sulphur signal runs perfectly parallel to the CeO signal, indicating that sulphur is only found in the GDC phase rather than in the YSZ substrate or Pt current collector. The curious peak-shaped increases of the CeO and ^34^S signal at the end of the interface region in Figure 3a are most likely due to shadowing effects of a part of the Pt current collector, which also explains the drawn out interface region. With a factor of about three, the reference measurement only shows a slightly increased sulphur signal in the GDC phase when compared to the substrate. In both cases, no diffusion profile is measured; on the contrary, the sulphur signal is constant throughout the whole depth of the 200 nm thick microelectrode.

Another interesting result of the ToF-SIMS experiments shown in Figure 3a,b, is that in both cases a strong Si signal is detected right at the surface of the composite microelectrode, which quickly attenuates deeper in the sample, apart from a small spike in the interface region. This silicon agglomeration on the surface is not entirely unexpected and has been hinted at in the literature [4,43]. Consequences for the electrochemical properties of the electrodes will be discussed below.

#### 3.1.2. Macroscopic Polycrystals

**Figure 4:**
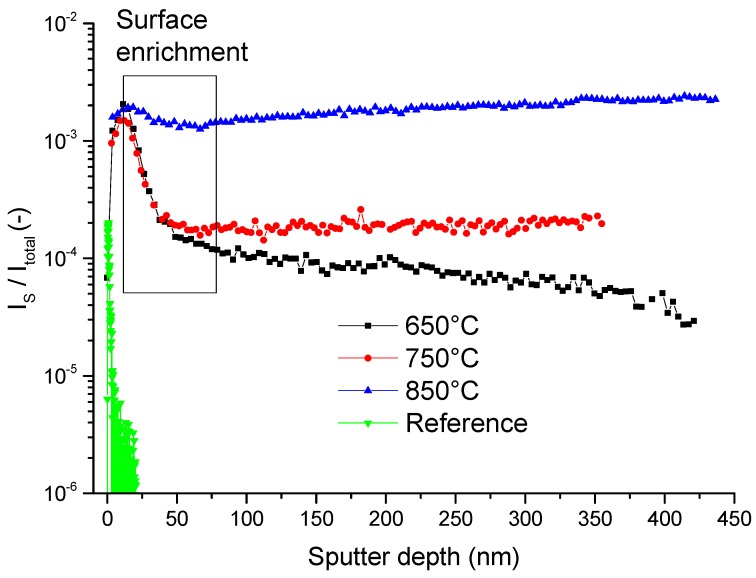
ToF-SIMS depth profiles of the normalized sulphur signal of GDC 10 polycrystals heat treated at the specified temperature in an atmosphere of dry 2.5% vol H_2_ in Ar for 72 h. As a reference sample an untreated GDC 10 polycrystal was used. The box highlights a surface enrichment region, which was fitted to Equation (1); the results are given in Table 2.

As the experiments on model composite electrodes in Figure 3 showed, sulphur is incorporated into GDC, but the diffusion coefficient at the monitored temperatures are too high to record a drop in the concentration profiles in the 200 nm thin layers. Therefore, in an attempt to measure diffusion coefficients of sulphur in GDC by means of ToF-SIMS, sulphur incorporation experiments were performed on macroscopic GDC 10 polycrystals over several days of exposure. To increase the rate of surface sulphur incorporation and to suppress possible back reactions with water [44,45], a dry hydrogen atmosphere was chosen. The resulting depth profiles are plotted in Figure 4. In all three samples, a significant increase of sulphur is detected in respect to a reference sample, which was not subjected to a sulphur containing atmosphere. Likewise, every sample, except the reference, shows a surface near enrichment region, highlighted with a rectangular box in Figure 4. The concentration profiles, assuming a lateral one-dimensional diffusion experiment from a constant source, can be described by Equation (1) [30,31].

The resulting surface incorporation constants, the diffusion coefficients and the diffusion times are listed in Table 2, with the results stemming from two sulphur isotopes measured simultaneously. Strangely, the extracted diffusion coefficients for the different temperatures are identical within the accuracy of the method. One might suspect surface space charges as a cause, however, in ceria their decay lengths are usually in the range of about 1 to 5 nm depending on dopant concentration [46,47]. On the other hand, the grain sizes of the samples are about 25 µm and therefore too large to explain the roughly 50 nm deep enrichment zone. Hence, it is reasonable to assume that the surface enrichment is caused by a different mechanism and profiles do not simply reflect sulphur bulk diffusion. Anticipating the results of Section 3.3, one might speculate that a phase change occurs at the surface. However, no such indications could be found by X-ray diffraction for the polycrystalline bulk samples presented in Figure 4.

Deeper in the sample the concentration drop tapers off for all three sulphur enriched samples. While the profile at the lowest temperature of 650 °C enters a region of slower decrease, the sample at the intermediate temperature of 750 °C reaches a plateau. Unexpectedly, for the sample treated at the highest temperature of 850 °C the concentration even increases slightly, which is hardly a real effect but most likely due to a SIMS artefact. Nevertheless, it is obvious that the normalized sulphur signal in the GDC bulk positively correlates with temperature. Judging from the decreasing trend in the bulk region of the sample annealed at 650 °C, the bulk profiles for all samples might in fact be small sections of even deeper diffusion profiles. The surprisingly high diffusivity of sulphur in the ceria lattice measured here has indeed been predicted in a very recent theoretical study, where it was found that the migration energy for a site exchange with an oxygen vacancy is actually very similar for sulphur and oxygen [48].

Analogous measurements were performed with samples that were covered with porous Pt paste. However, except a lower sulphur incorporation rate, likely due to the reduced free surface area, the results exactly mirror the results for the uncovered samples and are also found in Table 2.

### 3.2. X-ray Photoelectron Spectroscopy

**Figure 5:**
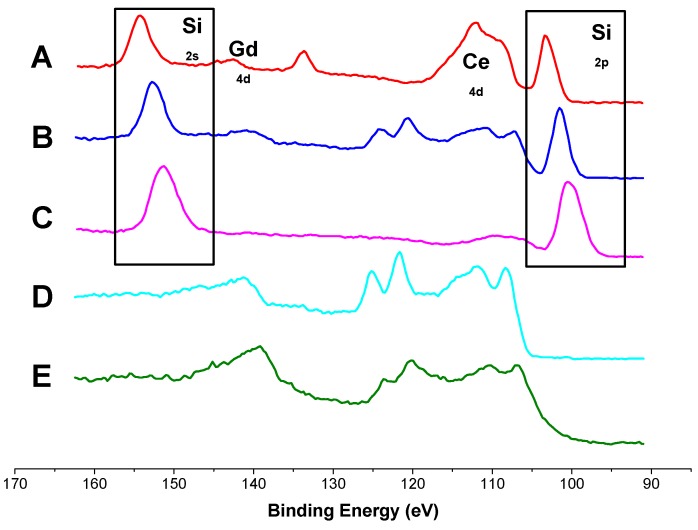
XPS spectra of the surface species of different GDC 10 samples treated at 750 °C in 2.5% vol H_2_ in Ar with a hydrogen to water ratio of 1:1 with intermediate exposure to a maximum of 10 ppm H_2_S. **A**: model-composite microelectrode asymmetrically heated for 150 h; **B**: model-composite microelectrode symmetrically heated for 51 days; **C**: model-composite microelectrode asymmetrically heated for 140 h; **D**: Pristine model-composite microelectrode after PLD preparation; **E**: Macroscopic GDC polycrystal. The boxes highlights signals associated with Si surface species, the rest of the peaks can be attributed to Ce or Gd.

XPS spectra of GDC composite layers recorded after different measurement times in wet hydrogen atmospheres are shown in Figure 5 along with data measured on a macroscopic GDC reference sample. Except for the reference sample E and the as-deposited layer D, every measured sample exhibits a strong peak associated with silicon on the GDC surface (highlighted with the boxes in Figure 5). These results agree very well with the findings of the ToF-SIMS study (see Figure 3a,b). No clear correlation between measurement times and silicon coverage is possible from the data available so far, as samples A and C were measured in an asymmetric heating stage, while sample B was symmetrically heated. No sulphur was detected on the surface in this XPS study, which is in agreement with literature [42] and SIMS results, see Section 3.1, as surface sulphur diffuses into the GDC bulk at elevated temperatures.

### 3.3. X-ray Diffraction

**Figure 6:**
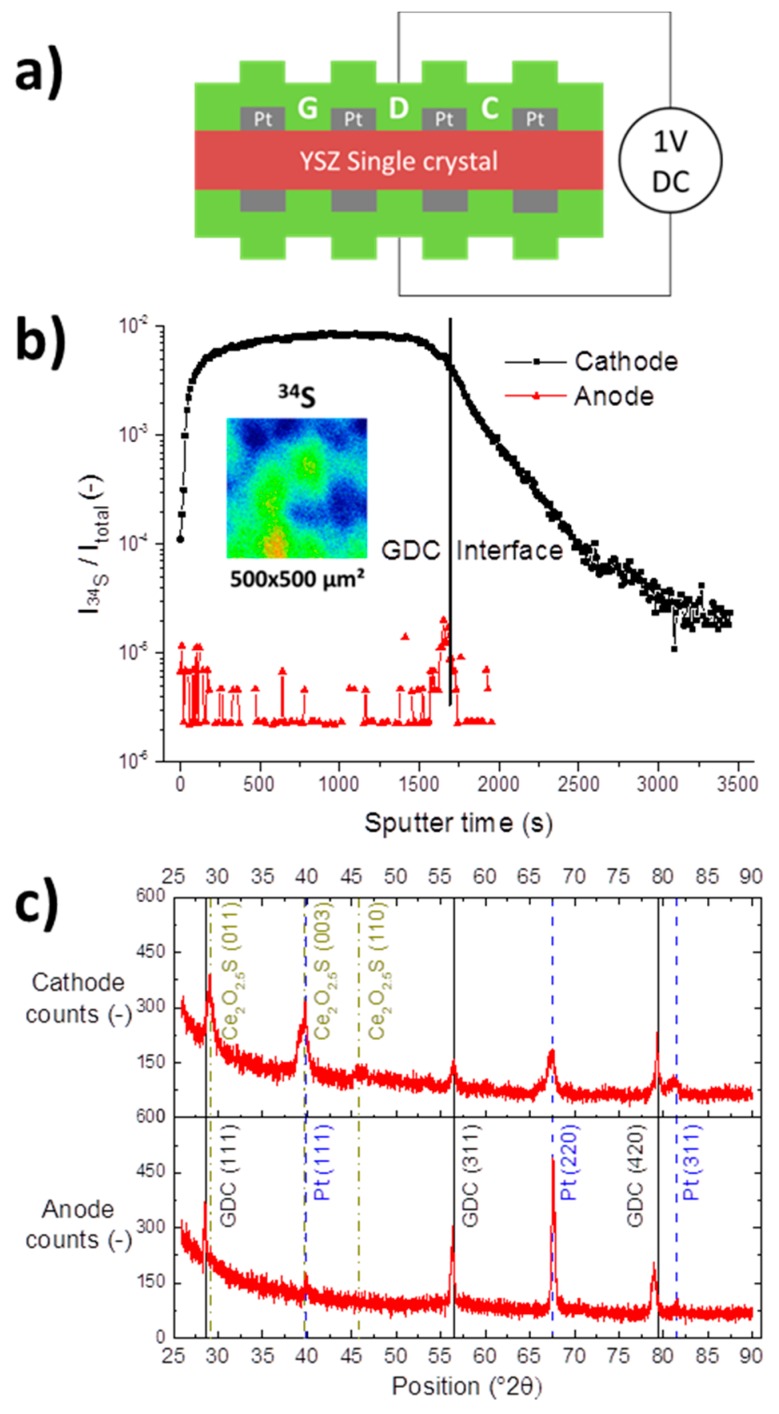
(**a**) Schematic cross sectional view and wiring of the electric sulphur implantation experiment. Both sides of a YSZ single crystal were covered with a model composite Pt/GDC 20 electrode layer and polarised for 48 h at 1 V at 750 °C in 2.5% vol H_2_ in Ar with a hydrogen to water ratio of 1:1; (**b**) ToF-SIMS depth profiles of the normalized ^34^S signal of the anode and cathode after the implantation step. The interface region refers to the overlap of Pt, GDC and YSZ signals as visualized in Figure 3c. The inset shows a top view ion image of the inhomogeneous lateral distribution of the ^34^S signal; (**c**) Gracing incidence X-ray diffraction patterns of anodic and cathodic Pt/GDC 20 layers. Reflexes associated with Pt, GDC 20 and Ce_2_O_2.546_S, a cerium oxysulphide phase, are highlighted.

A YSZ single crystal was coated on both sides with 200 nm GDC 20 layers with buried Pt current collectors. To enrich the GDC film with sulphur by cathodic polarisation, a constant voltage of 1 V was applied to the sample over two days in a humid hydrogen atmosphere with 10 ppm H_2_S, see sketch in Figure 6a. Assuming an equal polarisation resistance of cathode and anode, the applied voltage corresponds to an overpotential of about 400 mV at each electrode. The ToF-SIMS depth profile in Figure 6b shows a significant enrichment in sulphur in the cathode, proving a successful implantation step. In contrast, at the anode barely any sulphur signal is recorded. However, the sulphur signal at the cathode is not evenly distributed laterally as shown in the top view ion image, plotted as an inset in Figure 6b. Some spots appear to show a rather high sulphur incorporation activity, whereas other regions show less sulphur uptake.

In addition, both anode and cathode were characterized by gracing incidence X-ray diffraction (GID-XRD) and the obtained diffractograms are compared in Figure 6c. As expected, the anode and the cathode show peaks corresponding to GDC 20 and the Pt current collector. However, at the cathode side, additional signals were recorded, which could be matched to a hexagonal cerium oxysulphide phase, with Ce_2_O_2.546_S (powder diffraction file 01-087-0283) providing a marginally better match than Ce_2_O_2_S (powder diffraction file 00-026-1085). The Ce_2_O_2.546_S phase has so far not been reported in the context of SOFC research; however, Ce_2_O_2_S has been found at higher hydrogen sulphide concentrations [15,49]. Both compounds contain Ce^3+^ reduced from Ce^4+^ of the CeO_2_ fluorite lattice, additionally, XPS studies showed that adsorbed sulphur is incorporated into reduced ceria at elevated temperatures [42,50,51]. The sulphidation of ceria is described, e.g., in Refs. [12,19,52,53], as

(4) CeO2+12H2S+12H2⇄12Ce2O2S+H2O

Using the phase diagrams of the Ce-O-S system found in reference [49], the oxygen partial pressure at an overpotential of 400 mV at the anode of our symmetrical sample corresponds to the stability regime of CeO_2_, while at −400 mV on the cathode side Ce_2_O_2_S is the most stable species. Therefore, it is a reasonable assumption that it is indeed the increased Ce^3+^ concentration induced by the cathodic polarisation that facilitates the sulphur incorporation, with the inverse effect true for the anodic side.

### 3.4. Scanning Transmission Electron Microscopy

**Figure 7:**
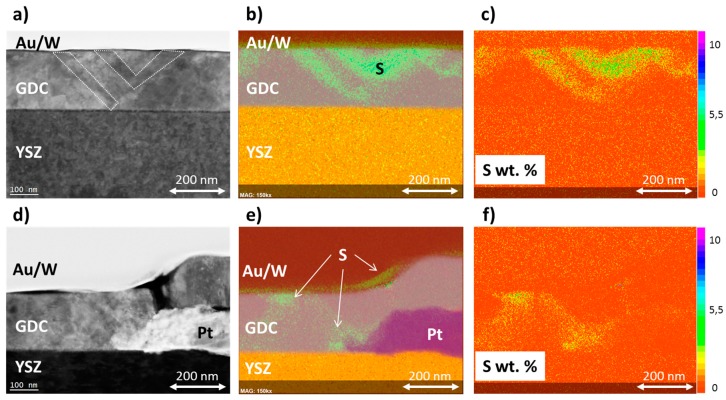
Scanning Transmission electron—angular dark field (STEM-ADF) micrographs and corresponding EDS elemental maps of a model-composite Pt/GDC 20 electrode, which was sulphur enriched by cathodic polarisation (see Figure 6). The gold and tungsten layers were applied as a protective cover during TEM preparation. (**a**) STEM-ADF micrograph of the GDC 20 layer away from Pt current collectors, the dotted areas correspond roughly to sulphur enriched zones; (**b**) EDS elemental maps of the investigated area in (**a**), the bright green areas in the GDC layer correspond to sulphur enriched regions; (**c**) Quantitative map (wt. %) of sulphur enriched regions in the part of the GDC layer imaged in (**a**,**b**); (**d**) STEM-ADF micrograph of the GDC 20 layer close to the Pt current collector and corresponding EDS elemental map in (**e**) and quantitative map of sulphur in (**f**), respectively.

The cathodically polarised sulphur enriched GDC 20 layer (see Figure 6) was further investigated by scanning transmission electron microscopy (STEM). Two different regions were imaged, one on the open GDC surface (see Figure 7a–c), and a second one closer to the Pt current collector away (see Figure 7e,f). In both cases, a thin near surface enrichment of sulphur is found in the corresponding energy-dispersive X-ray spectroscopy (EDS) maps in Figure 7b,e. However, by overlaying the signals of the Au/W cover layer with the Ce, Zr and Pt signals, it might be that this enrichment is located within the Au layer sputtered on the sample during TEM preparation. Furthermore, the contrast was very much enhanced to better visualize the sulphur enriched regions in the overlay images in Figure 7b,e, while in the quantitative sulphur maps in Figure 7c,f the surface near accumulation is much less pronounced. Nevertheless, areas of sulphur enrichment are also found within the GDC layer bulk, which definitively cannot be caused by an artifact during TEM sample preparation and, moreover, nicely mirror the result of the ToF-SIMS study in Figure 6b. These regions of high sulphur concentration penetrate the GDC layer like veins and follow features visible in the electron image, highlighted by the dotted areas in Figure 7a. From the quantitative EDS maps in Figure 7c,f, a maximum concentration of about 5 wt. % sulphur can be estimated in the enriched regions. These findings indicate that pathways with faster sulphur incorporation and diffusion exist within the GDC layer. It is known from the literature that the electron and, consequently, the Ce^3+^ concentration is increased at grain boundaries in ceria [46] and at ceria/YSZ interfaces [54], which might explain the sulphur enriched regions as speculated in Section 3.3. However, further and higher resolved images will be necessary to confirm this hypothesis.

### 3.5. BET and Sulphur Quantification

To quantify the sulphur uptake of GDC 10, a fine powder samples was annealed at 750 °C over 3 days in a wet hydrogen atmosphere with an H_2_S concentration of 10 ppm. A reference sample was also treated analogously in a sulphur free atmosphere. Afterwards, the surface area was determined by BET adsorption and the sulphur concentration by combustion and consecutive infrared quantification. 

The total amount of sulphur found in the combustion analysis would equate to a surface coverage of about 5%. However, the SIMS results presented in this study and XPS studies in literature [42,50,51] strongly suggest that the sulphur was incorporated into the GDC bulk. Assuming equally sized spheres, a mean powder size was calculated from the BET measurement. The results are given in Table 3. The half of the mean diameter of the powder of about 70 nm is well below the diffusion lengths probed in the ToF-SIMS studies presented in this work, see Figure 3 and Figure 4. Therefore, it is a reasonable hypothesis that the sulphur is evenly distributed in the grain volume leading to a concentration of roughly 350 mM. Assuming that sulphur is completely dissolved in the oxygen sub-lattice of cubic GDC, this amount corresponds to a situation where 0.2% of all oxygen sites are being occupied by a sulphur ion.

### 3.6. Impedance Spectroscopy

#### 3.6.1. Circular Composite Microelectrodes

**Figure 8:**
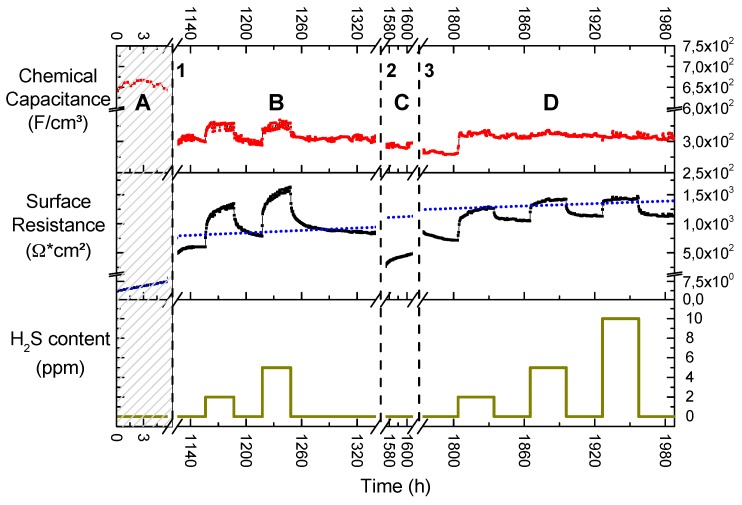
Evolution of chemical capacitance and surface resistance during a long-term measurement of a 300 µm diameter Pt/GDC 10 circular model-composite microelectrode at 750 °C. The atmosphere was 2.5% vol H_2_ in Ar with a hydrogen to water ratio of 1:1; the H_2_S content is plotted in the bottom diagram. In the shaded region, labelled **A**, the plotted data stem from a different electrode on the same but pristine sample. The data in regions **B**, **C** and **D** were all recorded on the same electrode. During the first break in the time-axis, labelled 1, the sample was exposed to 10 ppm H_2_S for a week. During break 2, the electrode was polarised at +0.2 V for 10 days. During break 3, measurements were paused for a week at room temperature. The dotted blue line in the resistance—time plot is an extrapolation of the linear degradation encountered on the pristine sample plotted in region **A**.

In Figure 8, the long-term evolutions of the chemical capacitance and the surface resistance of a circular GDC/Pt model-composite microelectrode exposed to different hydrogen sulphide concentrations are plotted. Since numerous microelectrodes are present on one sample, different experiments were conducted on the same sample but on different electrodes. Therefore, several axis interrupts are shown and explained in the figure caption.

##### Region A

The shaded area A represents data taken on the pristine sample, but on a different electrode than in the rest of the plot. While the chemical capacitance stays constant, the surface resistance shows very fast and linear degradation in the first few hours of measurement (please note the different scaling in *x*- and *y*-axes in the resistance plot in Figure 8). In the very beginning, the surface resistance was measured to be below 5 Ω·cm^2^, indicating a highly active GDC surface for hydrogen oxidation [4]. However, it degrades with a rate of roughly 0.7 Ω·cm^2^ per hour and its extrapolation is shown as a blue dotted line in the entire resistance plot. Experiments on other pristine samples showed a very similar behaviour with degradation rates ranging between 0.3 and 1 Ω·cm^2^ per hour. In accordance with the ToF-SIMS and XPS results this degradation behaviour can readily be explained by an increase of the Si coverage of the surface. The source of the Si might be the quartz parts of the setup [43], the GDC itself [4], or the YSZ electrolyte [55,56].

##### Region B

During the first axis break, labelled “1” in Figure 8, several experiments were conducted, including the hydrogen sulphide exposure for the SIMS study presented in Figure 3. This totalled to an exposure of 10 ppm H_2_S in the feed gas for about a week. However, despite this history the extrapolated surface resistance degradation from region A serves as a remarkably good estimate for the values measured several weeks later. This hints that the underlying degradation process is largely independent of the sulphide gas concentration and a very long-scale process at 750 °C in wet hydrogen atmospheres. This finding is again in agreement with the postulated Si poisoning, which is proposed as an explanation.

Upon exposure to H_2_S the surface resistance reacts with a fast almost step-like increase followed by an increased degradation rate of about 6 and 9 Ω·cm^2^/h for 2 or 5 ppm H_2_S, respectively. As soon as the H_2_S supply is switched off, there is again a step-like decrease in surface resistance followed by a slower recovery phase. Whether or not there is an additional—H_2_S-triggered—irreversible increase in surface resistance is hard to evaluate from the available data due to the long time scale of the processes at 750 °C. However, it is immediately obvious that there is no pronounced quantitative difference in the behaviour of the surface resistance between an exposure to 2 or 5 ppm H_2_S, except a slightly steeper step and marginally faster degradation for the higher concentration. Very similar behaviour was found in a study on sulphur sorbents based on ceria: The weight change of a ceria powder upon exposure to sulphur qualitatively mirrors the time dependence of the surface resistance in the present study [19]. Additionally, in a different study, it has been shown that the oxidation of methane on a GDC catalyst can be promoted by sulphur adsorption on ceria [53]. In both works the interaction of H_2_S with ceria was found to be subdivided into a fast and a slow process. The fast process has conclusively been ascribed to sulphur adsorption, while the slow process has been correlated with dissolution of sulphur into the bulk and possibly oxysulphide formation. In XPS studies dealing with hydrogen sulphide adsorption on reduced ceria thin films, it was found that at temperatures exceeding 600 K, only dehydrogenated sulphur is adsorbed to the surface and also bonds to surface oxygen vacancies [50], which excellently explains the fast increase in the surface resistance.

**Figure 9:**
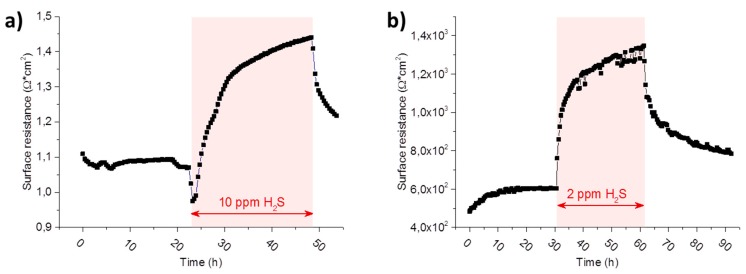
Long term evolution of the polarisation resistance of (**a**) a porous Ni/GDC 10 electrode at 780 °C normalized to the projected surface area and (**b**) a 300 µm diameter Pt/GDC 10 circular model-composite microelectrode of 200 nm thickness at 750 °C. The graph in (**b**) is a magnification of a feature in Figure 8 region B in the polarisation resistance plot. The atmosphere in (**a**,**b**) was 2.5% vol H_2_ in Ar with a hydrogen to water ratio of 1:1. The duration and the concentration of the hydrogen sulphide poisoning phases are highlighted in the graphs.

In Figure 9a the long term evolution of the polarisation resistance, normalized to the projected surface area, of a porous Ni/GDC 10 electrode in a wet hydrogen atmosphere at 780 °C is plotted. When comparing Figure 9a to Figure 9b, where an excerpt of region B in Figure 8 of a dense model composite electrode during a sulphur poisoning experiment is shown, it is immediately obvious how qualitatively similar both curves are. In studies on porous anodes, the resistance increase upon sulphur poisoning is sometimes ascribed to the metallic Ni current collector phase [11,57]. Additionally, changes in gas diffusion have also been suggested as a cause, due to a crystallographic phase transition and corresponding volume change in GDC [12], which would also have an effect on the polarisation resistance. However, due to the well-defined surface geometry and the absence of any metal/gas interfaces in the model composite electrode, such effects can be excluded here. Therefore, it is clearly shown here that the surface resistance of GDC also exhibits a pronounced sensitivity to H_2_S in the gas phase.

Between the pristine electrode data in area A in Figure 8 and the data shown in area B, the chemical capacitance exhibits a difference by a factor of about two. This difference might partly be explained by a different thickness of the two electrodes, caused by inhomogeneous pulsed laser deposition as indicated by a colour gradient visible across the sample. Additionally, impurity segregation to the grain boundaries [46] would take place during the initial stages of a long term measurement. This would cause a drop in total ionic conductivity and subsequently a reduced polarisation of the GDC layer above the Pt current collectors in the later stages of the experiment [25] (see also Section 3.6.2). However, besides this, the chemical capacitance stays remarkably constant during the whole measurement cycle, with marginally higher levels during H_2_S poisoning. The proposed sulphur surface coverage likely changes the concentration of Ce^3+^ and OH groups on the surface, which were identified to cause a surface-related capacitance on ceria in reducing conditions [37,58]. Additionally, the H_2_S premix is added as a dry gas into the humid hydrogen stream to avoid dissolution of the sulphide in the water of the humidifier and a slight drop in oxygen partial pressure is the consequence, which also leads to an increase in the chemical capacitance [37].

##### Region C

During the second axis break, labelled “2” in Figure 8, the sample was anodically polarised at 0.2 V for 10 days. The chemical capacitance is again unaffected by this treatment, but the surface resistance dropped by roughly a factor of 3. However, after a short nonlinear increase, the surface resistance settles on a degradation rate of 4 Ω·cm^2^ per hour. It has been demonstrated in Figure 6 that anodic polarisation protects GDC from sulphur incorporation. Indeed, it has been proposed that anodic current actively removes dissolved sulphur in doped ceria [12,20]. It is thus reasonable to assume that at least the surface region has been cleaned from absorbed sulphur by a combination of the long exposure to a H_2_S free atmosphere and the anodic bias. Accepting this interpretation, one could further speculate that the subsequent fast increase in resistance corresponds to a redistribution from sulphur in the GDC bulk to the surface, which has been reported in an XPS study on reduced ceria [51]. The extrapolated surface degradation from the pristine electrode, plotted as a blue dotted line in Figure 8, now overestimates the measured data; however, due to the previous polarisation treatment these values might not be comparable.

##### Region D

The last axis break, labelled “3” in Figure 8, marks pause for one week during which the sample was cooled to room temperature, after which the same electrode was reheated and measured further. The surface resistance starts with a slow decrease over 30 h, the absolute value of about 850 Ω·cm^2^ matches the downward sloping end of region B. This shows that the anodic polarisation caused no long-lasting improvement of the total surface resistance. The H_2_S poisoning phases once again show no proportionality between surface resistance increase and the sulphide concentrations. To the contrary, the poisoning effect plateaus on a similar level of about 1400 Ω·cm^2^, especially for 5 and 10 ppm H_2_S in the feed gas. Interestingly, this plateau is lower than for the maximum resistance during the poisoning experiments in region B of Figure 8. There is, however, a clear difference in the transient behaviour prior to reaching this plateau: The time to reach this resistance plateau increasingly shortens with higher H_2_S concentration and at 10 ppm almost no intermediate data points are resolved. After turning off the H_2_S supply, the surface resistance recovers and quickly reaches a plateau of about 1100 Ω·cm^2^. This behaviour is again in contrast to the experiments shown in region B. Interestingly, the extrapolated resistance degradation from the pristine electrode is again a good approximation to the actual measured surface resistance. However, given the comparatively short time the experiment lasted in region A of Figure 8, from which the data are extrapolated, this might as well be a coincidence at this stage in the long term experiment. The absence of a continuous slow degradation in H_2_S-containing atmosphere as well as the lack of the slow recovery phase after turning of H_2_S might be due to the fact that not many active surface regions remain due to the advanced state of Si-based degradation. While the adsorption effect, postulated for the results in region B, still exists, the amount of sulphur incorporated is probably minute and quickly diluted in the GDC bulk. Another interpretation might be that a kind of equilibrium is reached, most probably due to a solubility limit of sulphide in the GDC bulk at the respective H_2_S partial pressure, confer Equation (4), and no more bulk degradation is possible.

The chemical capacitance only reacts to the first H_2_S spike and stays constant during the rest of the measurement. Due to the advanced stage of degradation, a clear interpretation cannot be offered.

#### 3.6.2. Experiments on Composite Micro Electrodes with Interdigitated Current Collectors

The experiments on circular microelectrodes, shown in Figure 8, exhibited a multifaceted degradation and sulphur poisoning behaviour with several processes happening simultaneously. To probe more specific material parameter changes and facilitate a more detailed interpretation, microelectrodes with interdigitating current collecting fingers were employed, which allowed the measurement of chemical capacitance, surface resistance and, additionally, ionic and electronic conductivity [25].

##### Pristine Sample

**Figure 10:**
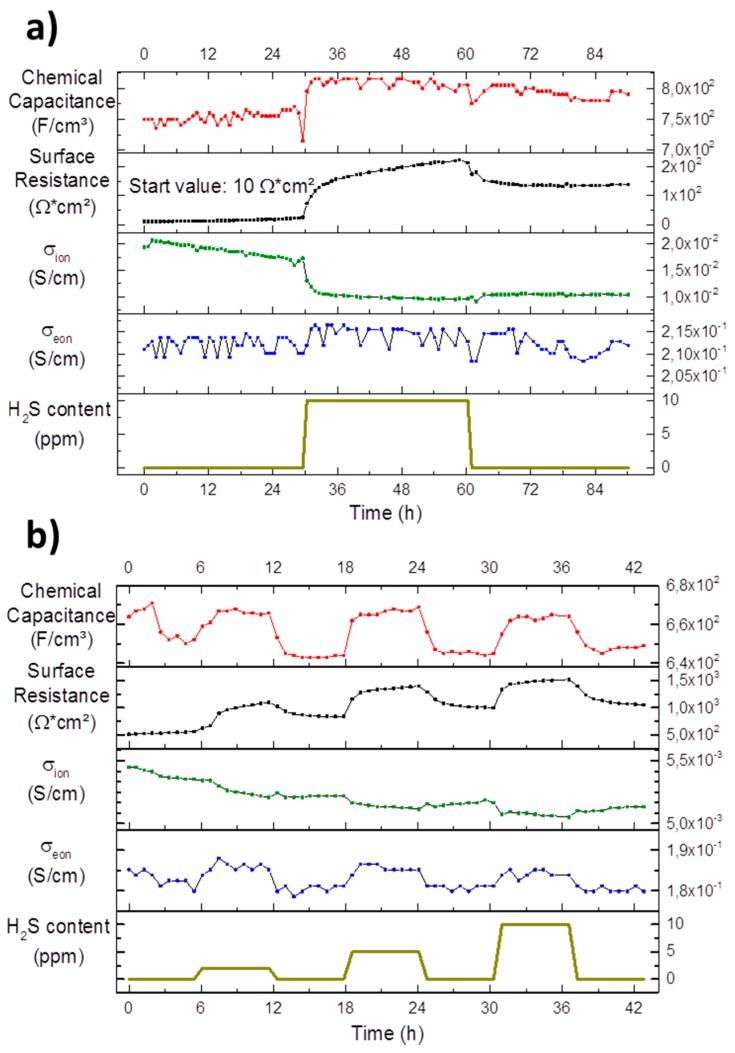
Chemical capacitance, surface resistance, ionic and electronic conductivity development of long-term measurements of Pt/GDC 10 interdigitated model-composite microelectrodes at 750 °C in an atmosphere of 2.5% vol H_2_ in Ar with a hydrogen to water ratio of 1:1; the H_2_S content in the feed gas is plotted in the bottom diagrams. (**a**) Measurements on a pristine sample. The interdigitated current collectors had a width 15 µm, a spacing of 15 µm and a length of 980 µm with a total of 8 digits; (**b**) Measurements on an aged electrode after two cool downs to room temperature and a photolithography step to improve contacting. The interdigitated current collectors had a width 15 µm, a spacing of 5 µm and a length of 980 µm with a total of 12 digits.

In Figure 10a, the fit results of a pristine GDC electrode are plotted. The surface resistance starts out low at about 10 Ω·cm_2_ with a degradation rate of 0.4 Ω·cm^2^/h. These values agree very well with the results from the pristine circular microelectrode shown in Figure 8. When the sulphur poisoning is started with 10 ppm H_2_S a sharp increase is again observed followed by an increased degradation rate of 2.5 Ω·cm^2^/h. However, in contrast to the microelectrode experiment, it is fairly obvious that an irreversible change in the surface resistance remains after the sulphide is removed from the gas phase. The surface resistance after the poisoning step stays constant at 150 Ω·cm^2^ over the measured time.

The absolute value of the chemical capacitance with 750 F/cm^3^ also matches the measurement on the pristine circular microelectrode in region A of Figure 8 with about 700 F/cm^3^. As before, it immediately reacts to the gas change at the beginning of the poisoning experiment with a sudden increase. After the poisoning experiment, the chemical capacitance seems to slowly decrease over the rest of the measurement.

The ionic conductivity of the GDC electrode plotted in Figure 10a shows a linear downward trend of 0.14 mS/(cm·h). Silicon is enriched on the surface according to the findings of the XPS results in Section 3.2. However, silicon is also known to accumulate in the grain boundaries of YSZ and GDC (46) forming an impurity phase there, which effectively hinders oxygen transport. While it is generally possible to separate the resistive properties of grain bulk and grain boundaries by electrochemical impedance spectroscopy, it is much more complicated in the thin film case [59,60]. Therefore, one can currently only speculate that this continuous decrease in ionic conductivity is indeed due to silicon segregating to the GDC grain boundaries. The first phase is followed by a step-like drop at the start of the sulphur poisoning and a further decrease of 0.03 mS/(cm·h). This decrease is not recovered at the end of the poisoning phase and the ionic conductivity reaches a plateau at about 10 mS/cm. It is speculated that the decrease upon sulphur exposition in ionic conductivity may be related to lattice distortions caused by incorporation of larger sulphur ions into the oxygen sub-lattice of the GDC host structure. The effect of lattice strain on oxygen transport is a highly active field, but out of scope of the present study. However, as a general rule, tensile strain tends to accelerate oxygen diffusion, while compressive strain, as would be expected in the vicinity of an incorporated sulphur ion, has the adverse effect [61].

In contrast, the electronic conductivity of the GDC electrode is constant at about 0.21 S/cm during the whole experiment, any possible trends are hidden beneath the data scatter. 

##### Aged Sample

In Figure 10b, the data of an aged electrode recorded on the same sample, as are the data shown in Figure 10a, are plotted, but after a cumulative week of operation at 750 °C with two cool downs to room temperature and an additional lithography step to improve contacting. The surface resistance and chemical capacitance exhibit qualitatively the same behaviour to sulphur poisoning as described for the pristine sample and for the circular microelectrode in region B in Figure 8. The absolute value of the surface resistance in excess of 1000 Ω·cm^2^ is tremendously higher than in the pristine sample, while, in contrast, the chemical capacitance has dropped slightly. These changes mirror the trends of the circular microelectrode data of Figure 8, when comparing the pristine electrode data in Region A to the rest of the plot.

While the ionic conductivity in Figure 10b exhibits a clear downward trend right at the beginning as well as during phases of sulphur poisoning with plateaus in between, it is important to note that the total change is about 5% and therefore very minute. The range of about 5 mS/cm is lower than the plateau at the end of the measurement of the pristine electrode in Figure 10a.

Finally the electronic conductivity in Figure 10b is again nearly constant, but follows the ups and downs of the chemical capacitance. This correlation can be explained straightforwardly, since the chemical capacitance is a direct measure of the concentration of electrons [37], which again linearly affects the electronic conductivity [62]. The better resolution of electronic effects on this electrode compared to the pristine one discussed above can be explained by the different geometry of the current collectors (for details see caption of Figure 9). The absolute value of the electronic conductivity of about 0.18 S/cm agrees very well with the values of the pristine electrode in Figure 10a, demonstrating only minor degradation effects of this material parameter.

The results from the impedance spectroscopy study on model-composites with interdigitating current collectors nicely show that the increase in polarisation resistance observed on circular microelectrodes during sulphur poisoning can almost exclusively be explained by a change in the surface exchange resistance. However, a slight but reproducible decrease of the ionic conductivity was also observed. In contrast, the electronic conductivity does not change with sulphur exposure or temperature cycling.

## 4. Summary of the Proposed Surface Resistance Degradation Mechanism

**Figure 11:**
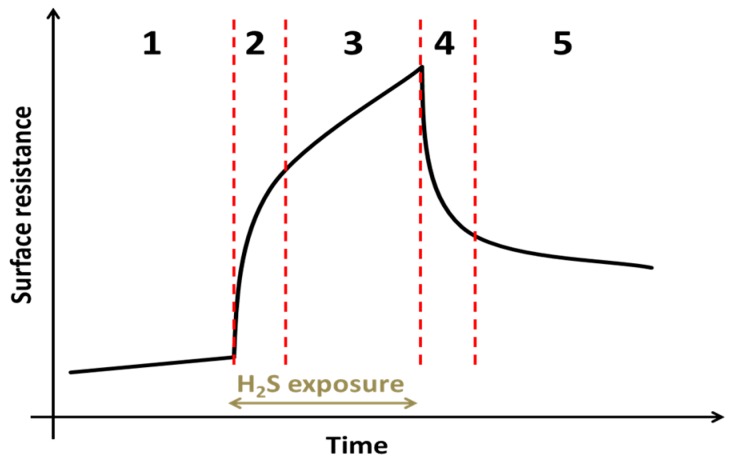
Schematic sketch of the surface resistance development of a GDC model composite electrode in reducing atmospheres with time during a typical sulphur poisoning experiment. Region **1**: Only slow degradation of the surface resistance is observed, attributed to Si poisoning. Region **2**: Upon the start of sulphur poisoning a fast increase of the polarisation resistance happens, which is most likely explained by surface adsorption of H_2_S and corresponding inhibition of the GDCs electrochemical active surface. Region **3**: Further decrease of the resistance potentially caused by adsorbed sulphur being incorporated into the GDC bulk. Region **4**: The sulphur supply is switched off and desorption of sulphur partly recovers the GDC surface. Region **5**: Slow recovery of the surface resistance, possibly connected to removal of absorbed sulphur in the GDC bulk.

The proposed degradation mechanism of the surface resistance of GDC model composite electrodes is summarized with the aid of the sketch in Figure 11: in Section 1, a constant degradation is observed due to the increasing surface coverage by a Si-species, either diffusing from the sample itself or transported there from the gas phase [4,43,55,56]. As soon as hydrogen sulphide is in the gas phase, a fast resistance increase happens in Section 2. This process is assumed to be related to an adsorption process of sulphide on GDC competitively inhibiting the reactive surface regions, such as oxygen vacancies [50]. Adsorbed sulphur is then slowly incorporated into the GDC bulk [42,50], which is speculated to be the main driver of degradation in Section 3. After the H_2_S supply is switched off, the surface sulphur species desorbs in Section 4, reversing the process in Section 2. In Section 5, the polarisation resistance slowly recovers from the bulk poisoning during the sulphur exposure. Whether or not a complete recovery can be achieved is still unclear and likely a function of temperature, H_2_S concentration as well as exposure and recovery time.

## 5. Conclusions

An array of analytical methods, including TEM, BET, XRD, ToF-SIMS, XPS, electrical impedance spectroscopy and combustion analysis, has been applied to characterize the interaction of hydrogen sulphide with gadolinia doped ceria in reducing atmospheres. It has been found by ToF-SIMS analysis that cathodic polarisation of model composite microelectrodes, consisting of a buried Pt current collector under a structured GDC layer on a YSZ single crystal, in an H_2_S containing atmosphere lead to a significant enrichment of the sulphur concentration within the microelectrode. Analogous experiments conducted on larger structures showed that this enrichment might even lead to phase changes, as measured by X-ray diffraction. Anodic polarisation effectively hampered sulphur incorporation.

ToF-SIMS showed an inhomogeneous lateral distribution of sulphur post cathodic incorporation. Subsequent analysis by transmission electron microscopy suggested that these regions of higher incorporation activity are related to the layer structure. Additionally, a high surface concentration of silicon was found on all layers by ToF-SIMS and XPS.

Diffusion experiments on macroscopic polycrystals yielded diffusion lengths of sulphur well beyond the probed volume of roughly 500 nm for an exposition of multiple days to hydrogen sulphide, at temperatures between 650 °C and 850 °C. However, higher temperatures are correlated with a higher bulk sulphur concentration. A temperature independent surface near enrichment of sulphur was also measured, but no definitive interpretation can be offered, although some hints are found that it might be related to a phase change to a cerium oxide sulphide.

BET adsorption in combination with combustion analysis allowed the quantification of the sulphur concentration in a GDC powder sample. The sulphur uptake after 3 days at 750 °C was around 0.016 wt. % or, assuming a homogeneous distribution in the bulk, 350 mM.

Electrochemical impedance spectroscopy studies on model-composite microelectrodes of circular shape and with interdigitated current collectors were conducted. It was found that material parameters associated with the bulk of the probed GDC layer, such as the chemical capacitance and the electronic conductivity, barely change over time and with exposition to hydrogen sulphide. The ionic conductivity showed a slight but consistent sensitivity to sulphur exposure, which was ascribed to lattice distortions due to the incorporation of the larger sulphur ion into the oxygen lattice of GDC. The surface related resistance, however, showed degradation even before exposure to hydrogen sulphide associated with the continuing contamination of the surface with Si impurities and might partially explain irreversible long term degradation effects. Furthermore, the polarisation resistance proved highly sensitive to H_2_S in the gas phase and showed a multi-facetted degradation over time. This behaviour has been tentatively explained by a combination of quick surface adsorption of a sulphur species, which leads to an obstruction of the reactive centres, and subsequent incorporation of sulphur in the surface near regions, leading to continuing degradation.

## Figures and Tables

**Table 1:** Comparison of material parameters of GDC 10 gathered on model-composite electrodes with interdigitated current collectors, cf. reference [25], to circular model-composite electrodes or literature values, respectively. The interdigitated current collectors had a width and spacing of both 30 µm and a length of 980 µm, cf. Figure 2. The current collector for the circular composite microelectrode was a grid of 10 µm width and 10 × 10 µm^2^ holes, cf. Figure 1a. Both current collectors were Pt buried beneath a 200 nm thick GDC layer. Measurements were performed at 750 °C in ~2.5% vol H_2_/~2.5% vol H_2_O/balance Ar. Literature values are averaged for measurements in dry 10% vol H_2_ in Ar and pure H_2_ with 3% vol H_2_O [35].

Parameter	Interdigitated Electrode	Reference Data	Source
C_chem_ (F/cm^3^)	330	292	Circular composite microelectrode, this study
R_surf_ (Ω·cm^2^)	21	7	
σ_ion_ (S/cm)	5.3 × 10^−2^	6 × 10^−2^	Extracted from reference [35]
σ_eon_ (S/cm)	4.2 × 10^−1^	1.2 × 10^−1^	

**Table 2:** Apparent diffusion coefficients *D* and surface incorporation constants *k* for sulphur incorporated into GDC 10 polycrystals in a dry ~2.5% vol H_2_ atmosphere in Ar with 10 ppm H_2_S for specified times and temperatures. *D* and *k*, as well as the background concentration *c_bg_* were evaluated using Equation (1) where applicable. For the uncovered sample at 857.7 °C, the background concentration was estimated manually.

Pt Coverage	Temperature (°C)	Diffusion Time (h)	Species	D (cm^2^/s)	K (cm/s)	c_bg_ (Counts/Total Counts)
No	649.8	67.23	^32^S	6.2 × 10^−18^	3.1 × 10^−14^	4.5 × 10^−5^
			^34^S	1.0 × 10^−17^	2.7 × 10^−16^	2.4 × 10^−6^
	749.4	68.65	^32^S	5.6 × 10^−18^	3.4 × 10^−14^	3.8 × 10^−4^
			^34^S	7.5 × 10^−18^	4.9 × 10^−16^	1.3 × 10^−5^
	857.7	42.68	^32^S	–	–	1.7 × 10^−3^
			^34^S	–	–	1.2 × 10^−4^
Yes	649.8	67.23	^32^S	6.0 × 10^−17^	2.5 × 10^−15^	3.6 × 10^−6^
			^34^S	1.5 × 10^−16^	1.1 × 10^−16^	1.2 × 10^−6^
	749.4	68.65	^32^S	4.7 × 10^−18^	4.6 × 10^−15^	1.4 × 10^−4^
			^34^S	4.5 × 10^−18^	1.5 × 10^−16^	1.6 × 10^−5^
	857.7	42.68	^32^S	7.7 × 10^−18^	2.0 × 10^−14^	8.0 × 10^−4^
			^34^S	8.0 × 10^−18^	8.0 × 10^−16^	6.0 × 10^−5^

**Table 3:** Specific BET surface areas, powder sizes and sulphur content of GDC 10 powder samples treated for 72 h at 750 °C in a wet 2.5% vol H_2_ in Ar atmosphere, with a ratio of hydrogen to water of 1:1. The powder size was calculated from the surface area by assuming equally sized spheres. The volumetric sulphur concentration assumes homogeneously distributed sulphur in the GDC bulk.

Gas Phase H_2_S Concentration (ppm)	BET Surface Area (m²/g)	Powder Size (nm)	Sulphur Content (wt. %)	Sulphur Concentration (mM)
0	6.114	135.2	< 3 × 10^−3^	< 68
10	5.947	139.0	1.6 × 10^−2^ ± 3 × 10^−3^	360
